# Role of *opuB* in Modulating Membrane Vesicle Composition and Function in *Streptococcus mutans* Under Neutral and Acidic Conditions

**DOI:** 10.3390/microorganisms13040884

**Published:** 2025-04-11

**Authors:** Wenyu Wang, Yiyi Huang, Huancai Lin, Yina Cao

**Affiliations:** 1 Hospital of Stomatology, Guanghua School of Stomatology, Sun Yat-Sen University, Guangzhou 510055, China; wangwy55@mail2.sysu.edu.cn (W.W.); huangyy99@mail2.sysu.edu.cn (Y.H.); 2 Guangdong Provincial Key Laboratory of Stomatology, Sun Yat-Sen University, Guangzhou 510055, China

**Keywords:** *Streptococcus mutans*, membrane vesicles, *opuB*, proteomics, lipidomics, acid stress

## Abstract

*Streptococcus mutans* (*S. mutans*) plays an important role in dental caries through acid production and biofilm formation. The membrane vesicles (MVs) of *S. mutans* are essential for microbial physiology, biofilm activity, and acid adaptation. The OpuB transporter regulates osmotic pressure in *Bacillus subtilis*; however, its role in *S. mutans* and its MVs remains unexplored. This study investigated the effects of the *opuB* pathway on MV biogenesis, as well as the proteomic and lipidomic profiles under neutral (pH 7.5) and acidic (pH 5.5) conditions. Nanoflow cytometry showed that the *opuB*-deficient strain (*Smu_opuB*) produced significantly more and smaller MVs than UA159 at pH 7.5, while the difference was not significant at pH 5.5. Lipidomic analysis revealed that *opuB* affected the lipid composition and concentration of *S. mutans* MVs. Proteomic analysis identified the differential enrichment of key metabolic processes associated with stress, including DNA repair. These findings highlight that *opuB* is an important regulator of MV biosynthesis and composition and may affect the environmental adaptability of *S. mutans* by regulating MVs.

## 1. Introduction

*Streptococcus mutans* (*S. mutans*) is a major pathogen of dental caries because it can tolerate an acidic environment and utilize carbohydrates to produce acid, leading to tooth surface demineralization [1,2,3]. *S. mutans* secretes membrane vesicles (MVs), which play important roles in intercellular communication and stress adaptation [4]. The co-culture of *S. mutans* and its MVs enhances biofilm development. Our previous research results showed that the MVs of *S. mutans* can be dispersed, and the adhesion-related proteins it carries can locally utilize sucrose to form extracellular polysaccharides [5]. The glucosyltransferases (GTFs) they carry may be released into the acquired pellicle. In the presence of sucrose, these enzymes locally hydrolyze sucrose to generate extracellular polysaccharides, including soluble and insoluble glucans, which facilitate bacterial adhesion to the tooth surface. Additionally, glucan-binding proteins (GBPs) on *S. mutans* can specifically interact with these polysaccharides, further promoting adhesion [6,7]. Even *S. mutans* MVs produced in an acidic environment can cause biofilm attachment [5]. In addition, *S. mutans* MVs can promote the biofilm formation of *Candida albicans*, thereby exerting their pathogenicity in the oral microecological system [8]. Beyond biofilm formation, *S. mutans* MVs have also been reported to activate mucosal immunity and induce specific antibody production against glucosyltransferases and PAc, similar to the immune-modulating effects observed in vesicles from *Staphylococcus aureus* and *Mycobacterium tuberculosis* [9,10,11]. The importance of the function of *S. mutans* MVs has been increasingly recognized, but little is known about its production. Therefore, elucidating the production mechanism of *S. mutans* MVs has important theoretical significance and clinical guidance value.

The OpuB ATP-binding cassette (ABC) transporter plays a crucial role in the osmoregulatory mechanisms of *Bacillus subtilis*, facilitating cellular adaptation to hyperosmotic stress by selectively accumulating compatible solutes such as glycine betaine and choline [12,13,14]. Structurally, OpuB comprises the following three conserved functional modules that operate in a coordinated manner: (i) the homodimeric ATPase subunit OpuBA, which provides energy for transport through ATP hydrolysis; (ii) the OpuBB/OpuBD transmembrane heterodimer, which forms the solute translocation channel; and (iii) the substrate-binding lipoprotein OpuBC, which initiates molecular recognition and uptake.

Upon exposure to elevated external osmotic pressure, OpuB is activated, triggering solute accumulation to stabilize intracellular osmotic balance and prevent cellular dehydration. This function is closely integrated with other transporters, such as OpuA and OpuC, which collectively regulate osmolyte uptake in response to specific stress conditions [15]. The interplays among these transport systems enable *Bacillus subtilis* to dynamically adjust its osmotic balance, enhancing its resilience and survival under fluctuating environmental conditions.

The formation of bacterial MVs is influenced by a range of environmental, chemical, and biological factors [16]. Abiotic stressors such as oxidative stress, UV radiation, pH fluctuations, temperature changes, osmotic pressure, desiccation, and hydration induce the production of MVs [17]. For example, acidic conditions and high osmotic pressure stimulate vesicle release as a protective response to expel harmful substances or release signaling molecules [18]. Other factors such as antibiotics, chemical treatments, phage effects, and bacteria–host interactions can also affect MV production [18].

OpuB transporter activation and MV production are ways for bacteria to cope with stressful environments; however, OpuB transporters’ influence on *S. mutans* MV biogenesis and composition has not been thoroughly investigated. We hypothesize that *opuB* regulates MV production and composition in *S. mutans* by modulating lipid remodeling and protein cargo selection under different pH conditions. Specifically, the deletion of *opuB* may disrupt membrane homeostasis, leading to altered MV lipid profiles, changes in MV size and abundance, and modifications in proteomic composition, which could influence bacterial adaptation to acidic environments and cariogenic potential. This study aims to elucidate the regulatory role of *opuB* by comparing MVs derived from *Smu_opuB* and the UA159 under neutral and acidic conditions. Understanding how *opuB* impacts MVs properties may provide insights into its role in *S. mutans* pathogenicity and survival in cariogenic environments.

## 2. Materials and Methods

### 2.1. Bacterial Strains and Growth Conditions

The experimental strains used in this study were *S. mutans* UA159 (ATCC^®^ 700610™) and *Smu_opuB* (Table 1). The standard strain, *S. mutans* UA159, was provided by the Institute of Microbiology, Guangdong Academy of Sciences (Guangdong Microbiological Analysis and Testing Center).

The *Smu_opuB* strain was constructed by knocking out the *opuB* gene in the UA159 chromosome using an erythromycin resistance cassette via polymerase chain reaction–ligation mutagenesis (Table S1). The successful construction of *Smu_opuB* was verified through sequencing (Figure S1).

The brain heart infusion (BHI) medium (Becton, Dickinson and Company, Sparks, MD, USA) was prepared by dissolving 3.7 g of BHI powder in 100 mL of ddH_2_O, heating the mixture to dissolve the powder, and allowing it to cool to room temperature. The pH was then adjusted to the desired values using 37% hydrochloric acid and NaOH powder. The medium was sterilized by autoclaving and stored in a sealed container at 4 °C until use.

Both strains were cultured in BHI medium at 37 °C under anaerobic conditions (80% N_2_, 10% CO_2_, and 10% H_2_). For maintaining selective pressure, erythromycin was added to the BHI liquid culture medium at a final concentration of 12.5 µg/mL [5].

We conducted the phenotypic characterization of both UA159 and *Smu_opuB* strains, including growth kinetic assays under both acidic (pH 5.5) and neutral (pH 7.5) conditions, along with a crystal violet staining assay to evaluate biofilm formation capacity (Figure S2).

### 2.2. Preparation of S. mutans MVs

The UA159 and *Smu_opuB* strains were cultured in 1 L of BHI broth until they reached the mid-log phase (OD_600_ = 0.2–0.3). The culture was then subjected to the following two centrifugation steps: the first at 6000× *g* for 15 min at 4 °C and the second at 10,000× *g* for 15 min at 4 °C to obtain the supernatant. The bacterial cells remaining in the supernatant were removed through membrane filtration (0.22 μm pore size, Millipore, Tullagreen, Ireland).

The supernatant was concentrated using a 100 kDa tangential flow filter (Millipore). After ultracentrifugation (100,000× *g*, 70 min, 4 °C; Beckman Coulter Type90Ti, Beckman Coulter, IN, USA.), the crude MVs underwent Optiprep density-gradient centrifugation (Sigma-Aldrich #D1556, St. Louis, MO, USA) in 10 mM HEPES-buffered solutions (pH 7.0). Specifically, 45% Optiprep (Sigma-Aldrich #D1556, St. Louis, MO, USA) (prepared by diluting 65% stock with 3% NaCl and 10 mM HEPES) was layered at the bottom of the tube, followed by sequential 500 μL layers of 40%, 35%, 30%, 25%, 20%, 15%, and 10% solutions. Post-centrifugation (200,000× *g*, 3 h, 4 °C; SW60 Ti rotor), the purified MVs were resuspended in PBS and stored at −80 °C. The supernatant was concentrated using a tangential flow filter with a 100 kDa cut-off (Millipore, Tullagreen, Ireland) [5].

### 2.3. Characterization of MVs

To confirm the presence and purity of the MVs, transmission electron microscopy (TEM) was performed following the protocol described [19]. Briefly, 10 μL of purified MVs were deposited onto a carbon-coated grid, negatively stained with 3% uranyl acetate, and washed with ddH_2_O. The grids were visualized using a Hitachi TEM System (Tokyo, Japan).

For MV characterization, nano-flow cytometry was conducted using a Flow NanoAnalyzer (NanoFCM Inc., Nottinghamshire, UK) equipped with 488 nm and 638 nm lasers. The system was calibrated with 250 nm Std FL SiNPs and silica nanospheres that were 68–155 nm in diameter for concentration and size, respectively. The MVs were diluted with PBS, and then the samples were loaded and analyzed following the manufacturer’s instructions. The particle concentration and size distribution of the MV samples (diluted in 0.22 μm pore size-filtered PBS) were calculated using the NanoFCM software (NF Profession V2.0), based on data collected over one minute under a sample pressure of 1.0 kPa [20].

### 2.4. Lipidomics Analysis

The lipid composition of the MV samples was analyzed using liquid chromatography–tandem mass spectrometry (LC-MS/MS). This analysis focused on identifying the lipid subclasses and their relative quantities in the MVs from both strains under the two pH conditions. In brief, the samples were subjected to lipid extraction with methanol, and the extracted lipids were dried under nitrogen gas. The lipids were then reconstituted with a 90% isopropanol/acetonitrile solution (*v*/*v* ratio of acetonitrile to isopropanol = 1:9) and injected into the LC-MS/MS system for analysis.

Reverse-phase chromatography was selected for LC separation using a Waters ACQUITY UPLC CSH C18 column (1.7 µm, 2.1 mm × 100 mm; Waters, DE, USA). The lipid extracts were re-dissolved in 200 µL 90% isopropanol (Thermo Fisher, Waltham, MA, USA)/acetonitrile (Thermo Fisher, USA), centrifuged at 14,000× *g* for 15 min using a low-temperature high-speed centrifuge (Eppendorf 5430R, Hamburg, Germany), and finally, 3 µL of the sample was injected. Solvent A was acetonitrile–water (6:4, *v*/*v*) with 0.1% formic acid and 0.1 mM ammonium formate, and solvent B was acetonitrile–isopropanol (1:9, *v*/*v*) with 0.1% formic acid and 0.1 mM ammonium formate. The initial mobile phase was 30% solvent B at a flow rate of 300 μL/min, held for 2 min, then linearly increased to 100% solvent B over 23 min, followed by equilibration at 5% solvent B for 10 min.

Mass spectrometry was performed using a Q-Exactive Plus mass spectrometer (Thermo Scientific, USA) in both positive and negative modes, coupled with a UHPLC Nexera LC-30A ultra-high performance liquid chromatograph (SHIMADZU, Kyoto, Japan). The ESI parameters were optimized as follows: source temperature, 300 °C; capillary temperature, 350 °C; ion spray voltage, 3000 V; S-Lens RF level, 50%; and scan range, *m*/*z* 200–1800 [21].

### 2.5. Proteomics Method

#### 2.5.1. Sample Preparation and Protein Extraction

The cells were lysed using SDT buffer (4% sodium dodecyl sulfate, 100 mM Tris-HCl (Thermo Fisher Waltham, MA, USA), 1 mM dithiothreitol, pH = 7.6) for protein extraction. Protein concentration was quantified using the bicinchoninic acid (BCA) Protein Assay Kit (Bio-Rad, Hercules, CA, USA).

#### 2.5.2. Protein Digestion

Protein digestion was performed using the filter-aided sample preparation (FASP) method, as described by Matthias Mann. Briefly, proteins were digested with trypsin, and the resulting peptides were desalted using C18 Empore™ SPE Cartridges (Sigma, St. Louis, MO, USA), with a bed I.D. of 7 mm and a volume of 3 mL. The desalted peptides were concentrated by vacuum centrifugation and reconstituted in 40 μL of 0.1% (*v*/*v*) formic acid [22].

#### 2.5.3. Liquid Chromatography–Tandem Mass Spectrometry (LC-MS/MS) Analysis

LC-MS/MS analysis was performed on a Q Exactive mass spectrometer (Thermo Scientific, Waltham, MA, USA) coupled with an Easy nLC 1200 chromatographic system (Thermo Fisher Scientific, Waltham, MA, USA). Peptide separation was achieved using a reverse-phase trap column (Thermo Scientific Acclaim PepMap100, 100 μm × 2 cm, nanoViper C18) connected to a C18 reversed-phase analytical column (Thermo Scientific Easy Column, 10 cm long, 75 μm inner diameter, 3 μm resin). The mobile phase consisted of buffer A (0.1% formic acid) and buffer B (84% acetonitrile and 0.1% formic acid). Peptides were separated with a linear gradient of buffer B at a flow rate of 300 nL/min, controlled by IntelliFlow technology. The mass spectrometer was operated in positive ion mode, using a data-dependent top 10 method to select the most abundant precursor ions for higher-energy C-trap dissociation (HCD) fragmentation. Survey scans were acquired with a resolution of 70,000 at *m*/*z* 200, and HCD spectra were obtained with a resolution of 17,500 at *m*/*z* 200. The automatic gain control (AGC) target was set to 3 × 10^6^, with a maximum injection time of 10 ms. Dynamic exclusion was applied with a duration of 40.0 s. The normalized collision energy was set to 30 eV, and the underfill ratio was set to 0.1% [22].

#### 2.5.4. Data Processing and Protein Identification

The MS raw data were processed using MaxQuant software (version 1.5.3.17) for peptide identification and quantification. Protein and peptide identifications were validated using a false discovery rate threshold of ≤0.01. Quantification was based on protein identification in at least two of three replicates for each group, with one group having null values for all proteins. Proteins were considered differentially abundant if they met the criteria of a *p*-value < 0.05 and a fold change >2.0, determined by a two-sided *t*-test.

#### 2.5.5. Functional Annotation and Enrichment Analysis

The protein sequences of differentially abundant proteins were queried against the NCBI BLAST+ database using the NCBI BLAST client (version 2.2.28+). Homologous sequences were identified using InterProScan (version 5.64-96.0), and gene ontology (GO) terms were assigned using Blast2GO software (version 2.5.0). GO term enrichment analysis was performed using R scripts (version 4.0.3), and only GO terms with adjusted *p*-values <0.05 were considered significantly enriched.

The differentially abundant proteins were further mapped to pathways in the Kyoto Encyclopedia of Genes and Genomes (KEGG) database using the KEGG orthology identifiers. Enrichment analysis was performed using Fisher’s exact test, with the full quantified proteome as the background dataset. The *p*-values were adjusted using the Benjamini–Hochberg correction for multiple testing, and pathways with adjusted *p*-values <0.05 were considered significantly enriched.

#### 2.5.6. Protein-Protein Interaction Network Analysis

Protein–protein interaction (PPI) networks for the differentially abundant proteins were constructed using STRING database (version 11.0). The results were visualized in Cytoscape (version 3.2.1), and the degree of each protein within the network was calculated to assess its centrality and functional importance.

### 2.6. Statistical Analysis

Data were analyzed using GraphPad Prism version 5.04 (San Diego, CA, USA). An unpaired *t*-test was applied to determine significant differences between the two groups. Statistical significance was defined as a *p*-value < 0.05. To ensure the accuracy and reproducibility of the results, each assay was performed in triplicate.

## 3. Results

### 3.1. Characterization of MVs

To assess the presence, size distribution, and concentration of MVs, we performed both TEM and nanoflow cytometry.

**Figure 1:**
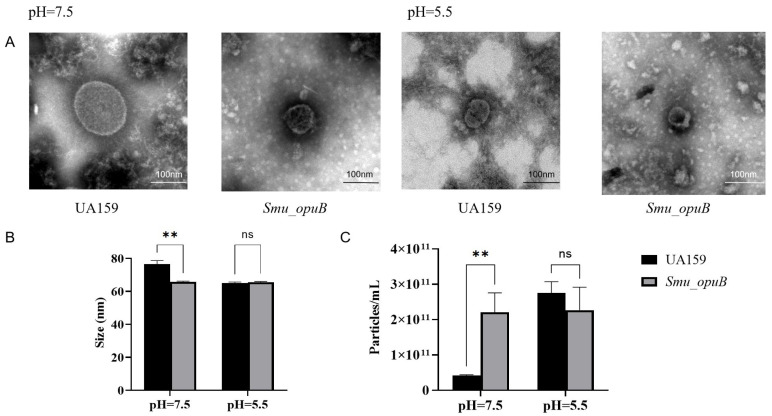
Characterization of MVs produced by *Smu_opuB* and UA159 under neutral and acidic conditions. (**A**) TEM images of MVs produced by *Smu_opuB* and UA159 under neutral and acidic conditions. (**B**) Size of MVs produced by *Smu_opuB* and UA159 under neutral and acidic conditions as measured by nanoflow cytometry. *n* = 3. (**C**) Concentration of MVs produced by *Smu_opuB* and UA159 under neutral and acidic conditions as measured by nanoflow cytometry. Data for all panels are expressed as mean ± S.D. ** *p* < 0.01, ns: no significant difference (Student’s *t*-test).

TEM imaging revealed the presence of MVs with typical spherical morphology and clear boundaries in both UA159 and *Smu_opuB* (Figure 1A and Figure S3). This morphology is consistent with the general characteristics of bacterial MVs, confirming their formation in both strains.

Nanoflow cytometry was used to determine the concentration and size distribution of MVs produced by *Smu_opuB* and UA159 under both neutral (pH 7.5) and acidic (pH 5.5) conditions (Figure 1B). At pH 7.5, *Smu_opuB* produced smaller (65.77± 0.47 nm) and a higher concentration of MVs (about 2.21 × 10^11^ ± 0.55 × 10^11^ vesicles/mL) compared to UA159 (about 76.5 ± 2.19 nm, 0.42 × 10^11^ ± 0.21 × 10^11^ vesicles/mL). However, under acidic conditions (pH 5.5), there was no statistical difference in concentration and particle size between the two.

### 3.2. Lipidomics Analysis of MVs

**Figure 2:**
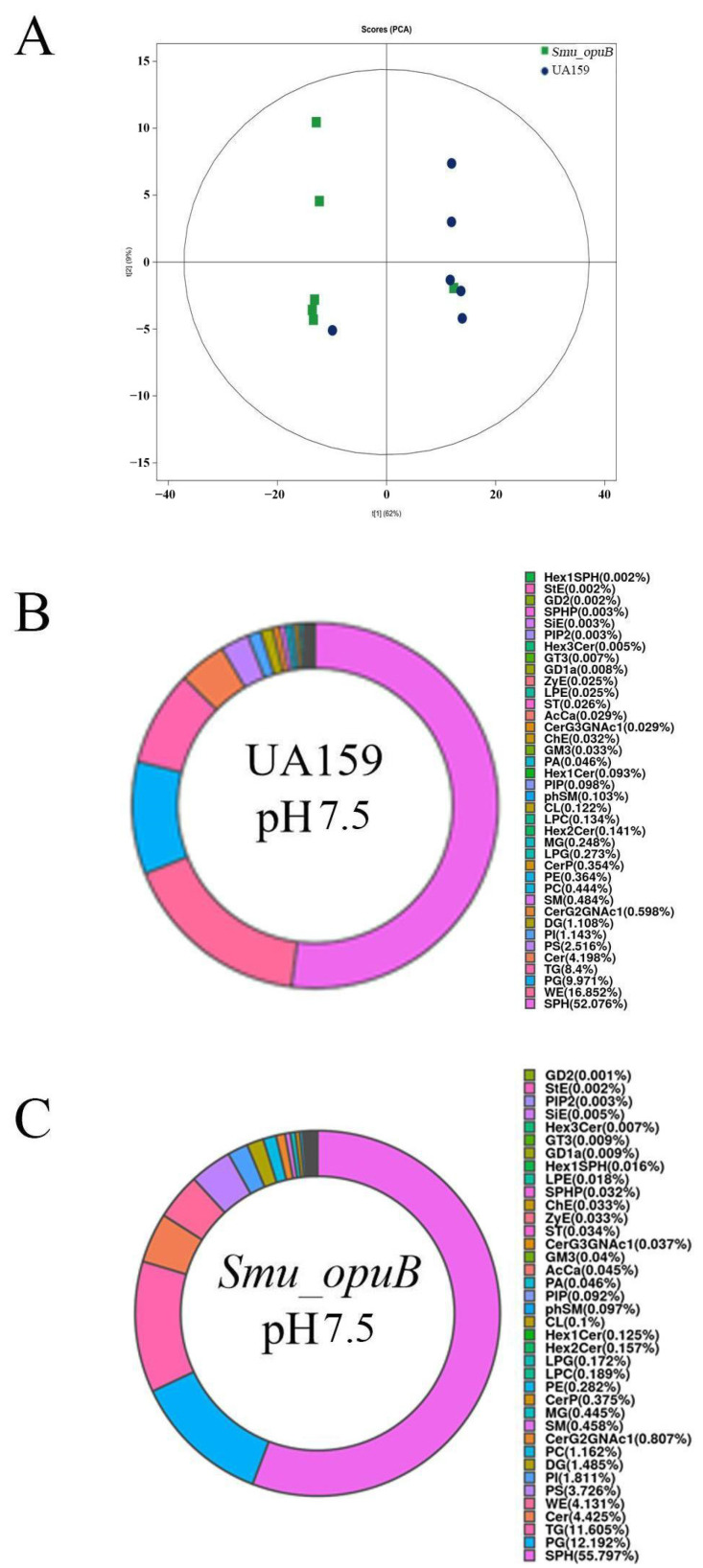
Lipidomic analysis of MVs produced by *Smu_opuB* and UA159 under neutral conditions. (**A**) PCA score plot of non-targeted lipidomics data. (**B**) Lipidomic profile (lipid classes) of MVs produced by UA159 under neutral conditions, shown as a pie chart. (**C**) Lipidomic profile (lipid classes) of MVs produced by *Smu_opuB* under neutral conditions, shown as a pie chart.

**Figure 3:**
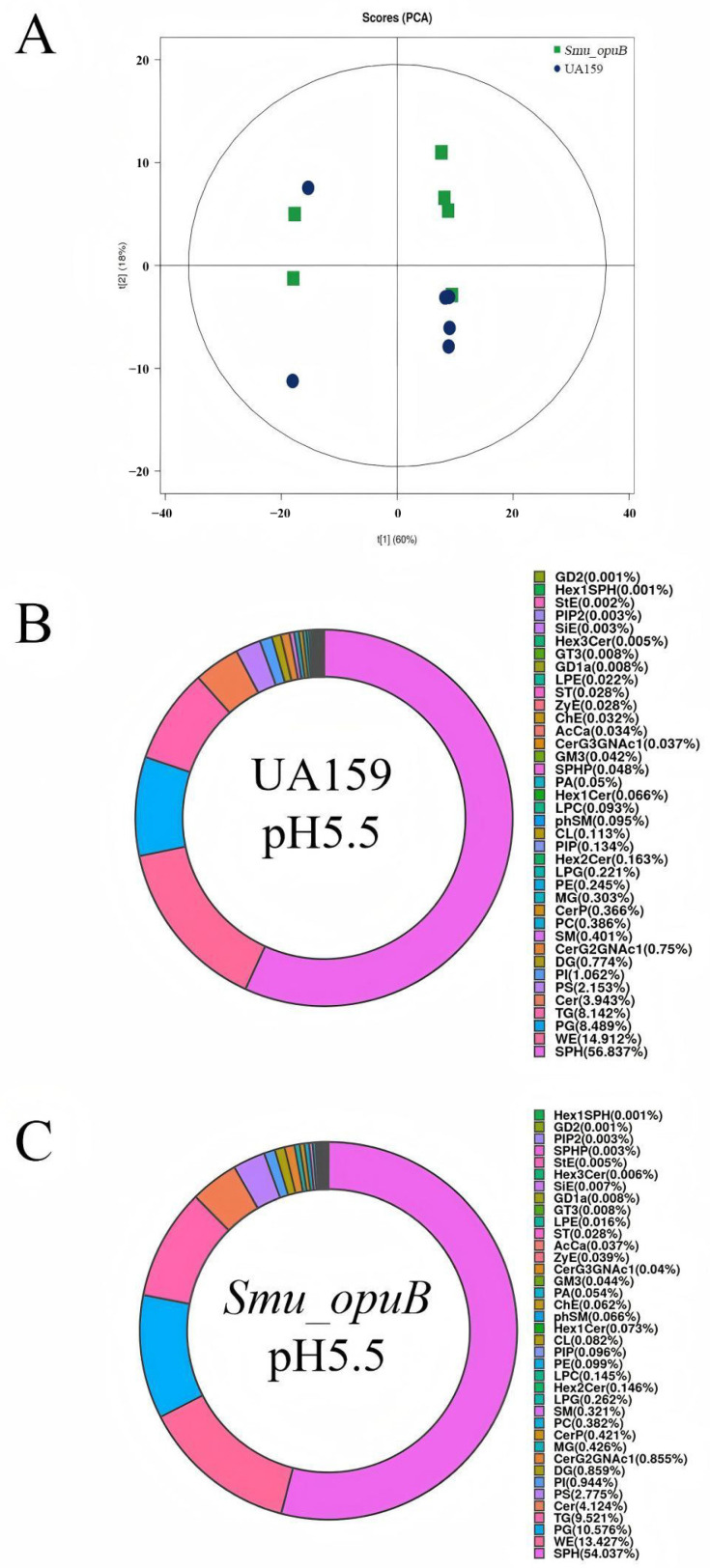
Lipidomic analysis of MVs produced by *Smu_opuB* and UA159 under acidic conditions. (**A**) PCA score plot of non-targeted lipidomics data. (**B**) Lipidomic profile (lipid classes) of MVs produced by UA159 under acidic conditions, shown as a pie chart. (**C**) Lipidomic profile (lipid classes) of MVs produced by *Smu_opuB* under acidic conditions, shown as a pie chart.

To evaluate whether the knockout of *opuB* affects lipid metabolism in *S. mutans* MVs, lipidomic analyses were performed on the MVs from *Smu_opuB* and UA159 formed at pH 5.5 and 7.5. Non-targeted lipidomic sequencing was used to investigate changes in lipid composition. Principal component analysis (PCA) score plots of non-targeted lipidomic data validated the confidence of sequencing data (Figure 2A and Figure 3A). Based on the pie charts generated from these data, lipid composition was altered after *opuB* knockout (Figure 2B,C and Figure 3B,C). The results showed that there were no significant differences in the chain length and lipid unsaturation of the MVs formed by the two strains under neutral or acidic conditions (Figures S4–S7). However, we observed significant changes in the concentrations of certain lipid subclasses, such as monoglycerides (MGs) and acylcarnitines, which may reflect different MV functions and stress responses under these conditions.

#### 3.2.1. Lipid Subclass Concentration at pH 7.5

**Figure 4:**
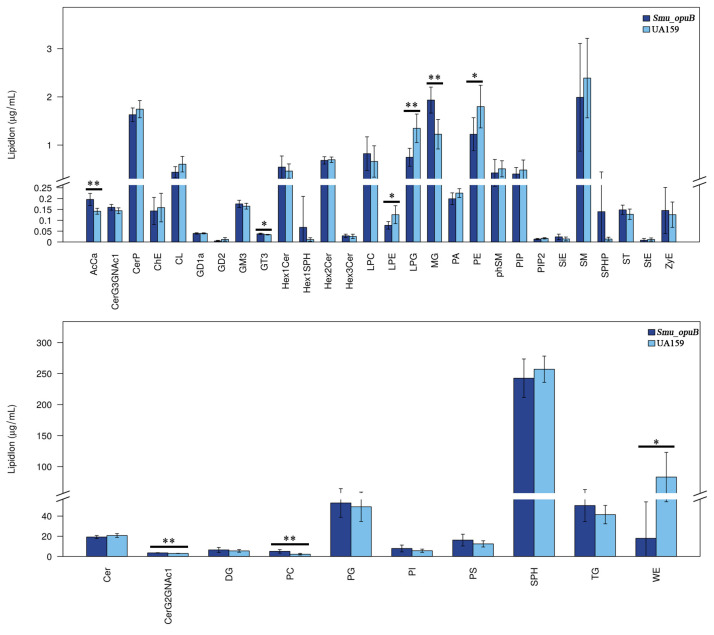
Significant differences in lipidomic profiles (lipid class) between MVs produced by *Smu_opuB* and UA159 under neutral conditions (selected VIP total >1 and *p* value < 0.05). *n* = 6. Data are expressed in all panels as mean ± S.D.: * *p* < 0.05, ** *p* < 0.01 (Student’s *t*-test).

The concentrations of acylcarnitine (AcCa), ceramide G2 glycans (CerG2GNAc1), monoglyceride (MG), and phosphatidylcholine (PC) were significantly higher in the *Smu_opuB* MVs (Figure 4). Acylcarnitine is involved in fatty acid transport and membrane remodeling, ceramide plays a role in membrane integrity and signal transduction, and phosphatidylcholine is the main structural component of cell membranes.

The levels of lysophosphatidylethanolamine (LPE), Lysophosphatidylglycerol (LPG), and phosphatidylethanolamine (PE) were significantly reduced in the *Smu_opuB* MVs (Figure 4). In addition, a significant decrease in the concentration of wax esters (WE) was observed in the *Smu_opuB* MVs (Figure 4).

#### 3.2.2. Lipid Subclass Concentrations at pH 5.5

**Figure 5:**
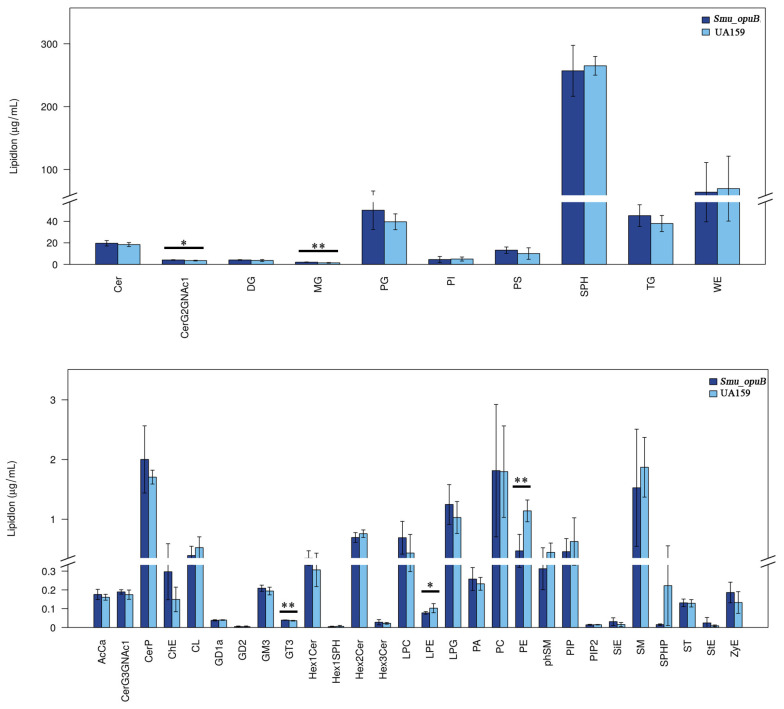
Significant differences in lipidomic profiles (lipid class) between MVs produced by *Smu_opuB* and UA159 under acidic conditions (selected VIP total >1 and *p* value < 0.05). *n* = 6. Data for all panels are expressed as mean ± S.D. * *p* < 0.05, ** *p* < 0.01 (Student’s *t*-test).

At pH 5.5, the *Smu_opuB* MVs had higher levels of CerG2GNAc1, slightly increased concentrations of glycosyltransferase 3 (GT3), and significantly higher levels of monoglycerides (Figure 5).The contents of LPE and PE were significantly reduced in the *Smu_opuB* MVs (Figure 5).

### 3.3. Proteomics of MVs

#### 3.3.1. Proteomics Analysis of Differential Protein Expression

Label-free proteomic analysis was conducted to identify and quantify proteins whose abundances significantly differed between the *Smu_opuB* and UA159 MVs under neutral (pH 7.5) and acidic (pH 5.5) conditions.

**Figure 6:**
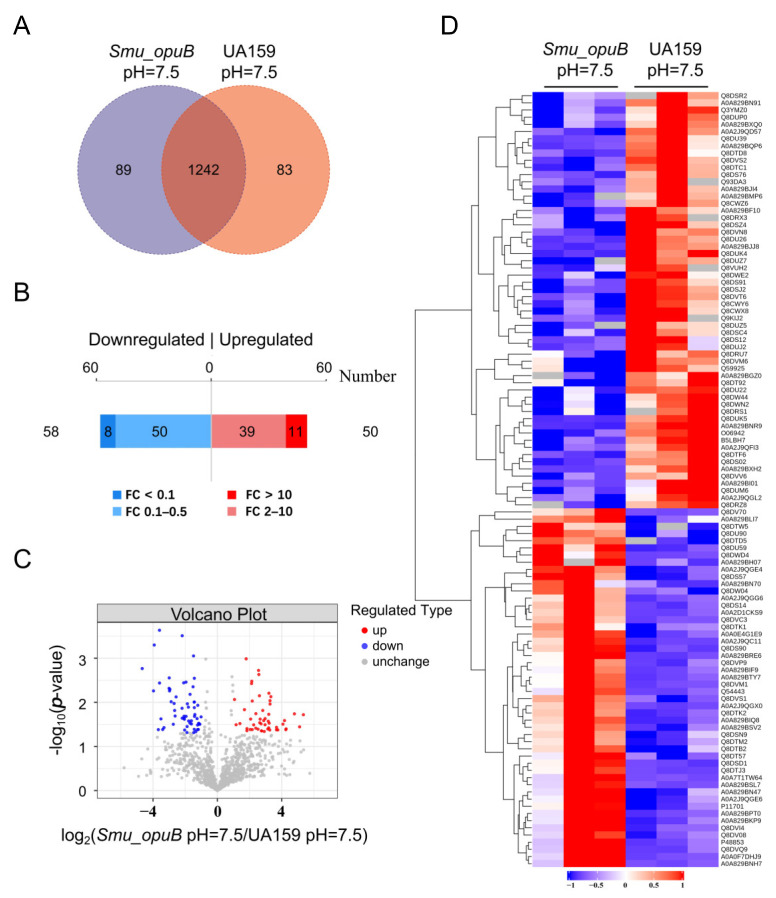
Proteomic analysis of MVs produced by *Smu_opuB* and UA159 under neutral conditions. (**A**) Venn diagram of proteins in the different groups under neutral conditions. (**B**) 108 proteins were significantly differentially abundant under neutral conditions. (**C**) Volcano plot indicating the significantly differentially abundant proteins between *Smu_opuB* and UA159 MVs under neutral conditions. (**D**) K-means clustering heatmaps between the *Smu_opuB* and UA159 MVs under neutral conditions.

As a result, under neutral conditions, 1242 proteins were identified to coexist in the *Smu_opuB* and UA159 MVs. A comparative analysis revealed 89 proteins uniquely identified in the *Smu_opuB* MVs and 83 proteins specific to the UA159 MVs, based on detection thresholds of the analytical platform. The Venn diagram of proteins in the different groups is shown in Figure 6A. Based on a fold change of >2 or <0.5 (with *p*-value set at <0.05), opuB deficiency resulted in changes in 108 proteins. Among them, 50 proteins were upregulated, and 58 proteins were downregulated (Figure 6B). A volcano plot indicated the significant differences in proteins between the (Figure 6C). We also identified significant changes between two groups using K-means clustering heatmaps. This analysis revealed good consistency among the parallel samples in the group, and the protein expression difference was obvious between the *Smu_opuB* and UA159 MVs (Figure 6D).

**Figure 7:**
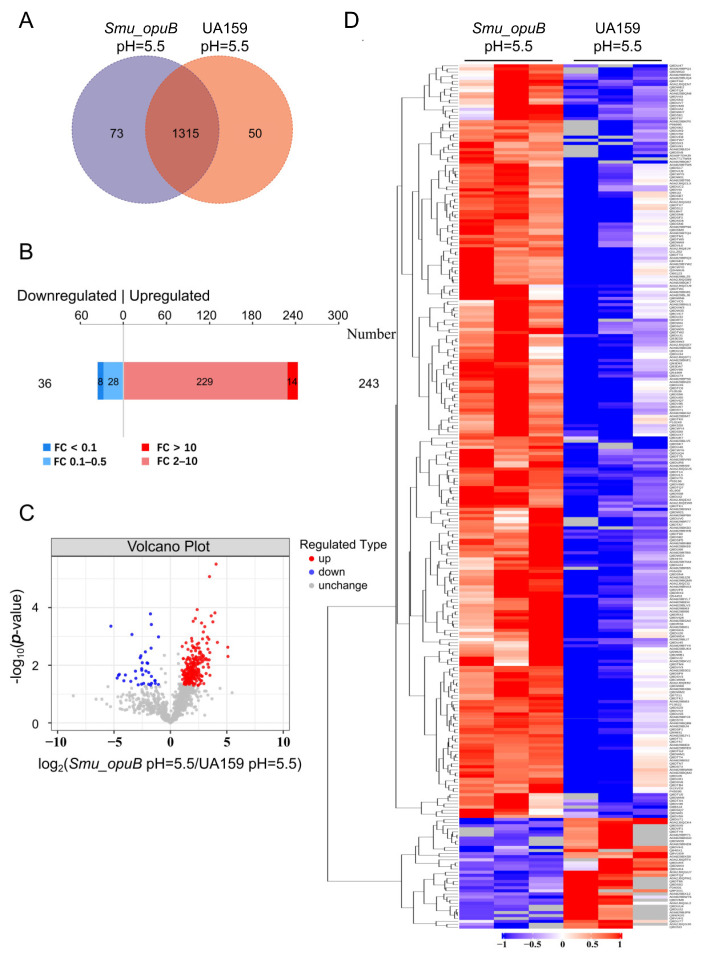
Proteomic analysis of MVs produced by *Smu_opuB* and UA159 under acidic conditions. (**A**) Venn diagram of proteins in different groups under acidic conditions. (**B**) 279 proteins were significantly differentially abundant under acidic conditions. (**C**) Volcano plot indicating the significantly differentially abundant proteins between *Smu_opuB* and UA159 MVs under acidic conditions. (**D**) K-means clustering heatmaps between *Smu_opuB* and UA159 MVs under acidic conditions.

Under acidic conditions, 1315 proteins were identified to coexist in the *Smu_opuB* and UA159 MVs. Compared with the control, 73 proteins were exclusively detected in the *Smu_opuB* MVs, while 50 proteins were only detected in the UA159 MVs. The Venn diagram of proteins in the different groups is shown in Figure 7A. Based on a fold change of >2 or <0.5 (with *p*-value set at <0.05), opuB deficiency resulted in changes in 279 proteins. Among them, 243 proteins were upregulated, and 36 proteins were downregulated (Figure 7B). A volcano plot indicated the significant differences in proteins between the *Smu_opuB* and UA159 MVs (Figure 7C). We also identified significant changes between the two groups using K-means clustering heatmaps. This analysis revealed good consistency among the parallel samples in the groups, and differences in the protein expression were obvious between the *Smu_opuB* and UA159 MVs (Figure 7D).

In the screening of significantly different proteins, we applied the criteria of a fold change (FC) >2 times (upregulation greater than 2 times or downregulation less than 0.50 times) and a *p* value < 0.05. The supplemental data include two comprehensive datasets (Tables S2 and S3) detailing the 20 most differentially abundant proteins identified through quantitative proteomic analysis.

#### 3.3.2. Functional Annotation and Pathway Analysis

GO ontology enrichment was applied to classify proteins in terms of their involvement into three main categories (biological process, molecular function, and cellular component) and the enriched GO terms (top 20) proteins were used to describe each classification.

**Figure 8:**
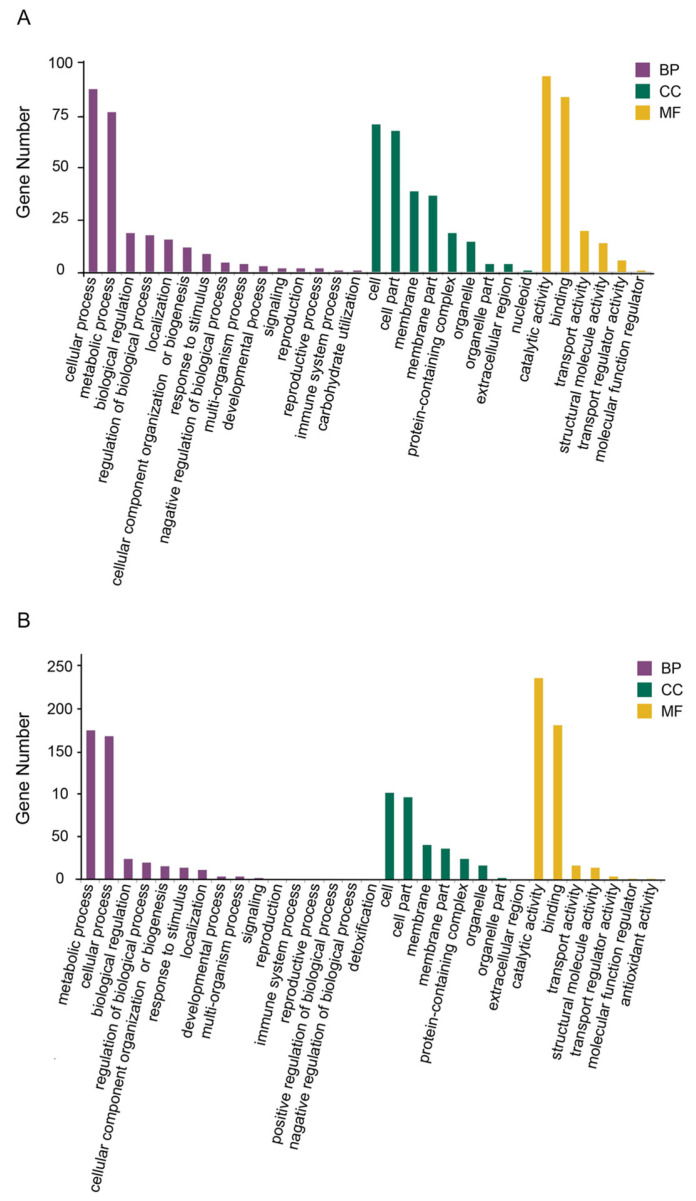
GO annotation analysis. (**A**) Enriched GO Terms (top 20) associated with the proteins of MVs produced by *Smu_opuB* and UA159 in neutral environment. (**B**) Enriched GO Terms (top 20) associated with the proteins of MVs produced by *Smu_opuB* and UA159 in acidic environment.

In a neutral environment, most of the proteins, according to the biological process classification, were associated with cellular and metabolic processes. In the molecular function category, proteins related to catalytic activity and binding occupied the largest parts. The cellular component was mainly occupied by cell, cell part, membrane, membrane part and protein-containing complex (Figure 8A).

In an acidic environment, most of the proteins, according to the biological process classification, were associated with metabolic and cellular processes. In the molecular function category, proteins related to catalytic activity and binding occupied the largest parts. The cellular components were mainly occupied by the cell, cell part, membrane, membrane part, and protein-containing complex (Figure 8B).

**Figure 9:**
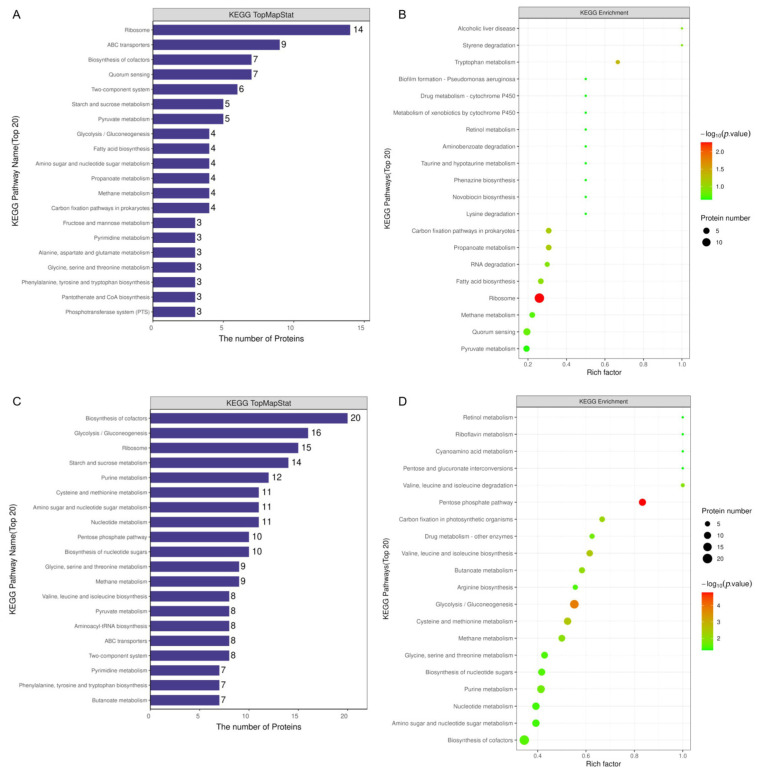
KEGG pathway analysis. (**A**) KEGG pathway names (Top 20) and number of the proteins of MVs produced by *Smu_opuB* and UA159 at neutral environment. (**B**) KEGG pathway names (Top 20) and the rich factor, proteins of MVs produced by *Smu_opuB* and UA159 in a neutral environment. (**C**) KEGG pathway names (Top 20) and number of the proteins of MVs produced by *Smu_opuB* and UA159 at acidic environment. (**D**) KEGG pathway names (Top 20) and the rich factor, proteins of MVs produced by *Smu_opuB* and UA159 in an acidic environment.

The top 20 KEGG pathways were mapped. In a neutral environment, ribosomes included the greatest number of proteins (14 proteins), followed by ABC transporters (9 proteins), biosynthesis of cofactors (7 proteins), quorum sensing (7 proteins), and two-component system (6 proteins) (Figure 9A). Among these, two pathways were enriched in terms of their effect on adapting to a neutral environment, including ribosome and quorum sensing. The detailed information for the KEGG enrichment is presented in Figure 9B.

In an acidic environment, the biosynthesis of cofactors included the greatest number of proteins (20 proteins), followed by glycolysis/gluconeogenesis (16 proteins), ribosomes (15 proteins), starch and sucrose metabolism (14 proteins), and purine metabolism (12 proteins) (Figure 9C). Among these, glycolysis/gluconeogenesis were enriched in terms of their effect on adapting to an acidic environment. The detailed information for the KEGG enrichment is presented in Figure 9D.

#### 3.3.3. Protein–Protein Interaction Network

**Figure 10:**
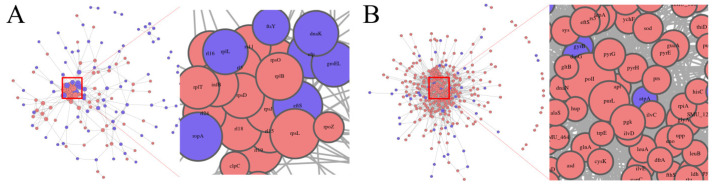
The PPI network analysis. (**A**) PPI network of the proteins of MVs produced by *Smu_opuB* and UA159 in a neutral environment. (**B**) PPI network of the proteins of MVs produced by *Smu_opuB* and UA159 in an acidic environment.

A protein-protein interaction (PPI) network was constructed to investigate the potential functional connections between the DEPs. At pH 7.5, a total of 439 interactions were identified between 180 proteins, with the most notable interactions involving eftS, rpsL, ropA, and rl19 (Figure 10A). At pH 5.5, a total of 1524 interactions were identified between 311 proteins, with the most notable interactions involving polI, purL, and glyA, which were connected to a broad network of stress response and metabolic proteins (Figure 10B). The analysis revealed that polI interacted with the largest number of proteins (118 interactions), followed by purL (71 interactions) and glyA (50 interactions), indicating their central roles in *S. mutans*’ response to environmental changes.

## 4. Discussion

This study comprehensively analyzed how *opuB* affects MVs biogenesis and composition in *S. mutans*, especially under different pH conditions. The combined proteomic and lipidomic analyses allowed us to gain a deeper understanding of the mechanisms by which *S. mutans* adapts to the acidic environment and the role of *opuB* in it. Our results suggest that *opuB* plays an important role in regulating the production, size, and composition of MVs, which is essential for the bacteria to adapt to the changing pH environment of the oral cavity.

In this study, we found an interesting phenomenon; under neutral conditions, *Smu_opuB* produced more MVs and smaller sizes compared with UA159. Other researchers have also found that some bacteria can form more and smaller MVs under certain stress conditions [23]. Combined with the proteomics data for analysis, we believe that the smaller MV size produced by *Smu_opuB* under neutral conditions (pH 7.5) may indicate that the MV formation process is more efficient or active. MVs play a crucial role in bacterial communication, stress response, and pathogenesis, and the increased production of smaller MVs may enhance the ability of *Smu_opuB* to quickly respond to environmental signals or challenges such as nutrient limitation or antimicrobial agents [24,25]. Smaller MVs also tend to have a higher surface area to volume ratio, which may facilitate interactions with host tissues or biofilm formation.

Under acidic conditions, there were no significant differences in the MV concentration and size between the two strains, which may be due to the activation of alternative stress response pathways that compensate for the knockout of *opuB*. Our proteomics results also showed that multiple stress response pathways were upregulated under acidic stress, and the differential proteins showed higher DEP connectivity in the protein interaction network, which suggested that both strains may operate at their maximum stress response capacity under acidic conditions, thereby minimizing the effect of *opuB* knockout on MV production.

Our proteomics results also showed that multiple stress response pathways were upregulated under acidic stress, and the differential proteins showed higher DEP connectivity in the protein interaction network, which suggested that both strains may operate at their maximum stress response capacity under acidic conditions, thereby minimizing the effect of OpuB loss on MV production.

Our lipidomics data showed that the lipid composition and concentration of *Smu_opuB* MVs, such as phospholipids and fatty acids, were significantly altered compared to UA159 secreted MVs under both then neutral and acidic conditions.

Under the neutral conditions, the phospholipid concentration in the MVs of *Smu_opuB* was slightly increased compared to UA159, and the distribution of specific phospholipid species was significantly changed. However, under acidic conditions, the differences in phospholipid concentrations became more pronounced, and the *Smu_opuB* MVs showed more significant increases in certain phospholipid species. This suggests that the OpuB transporter may be involved in regulating membrane lipid synthesis in response to acid stress, thereby potentially enhancing membrane stability under acidic conditions.

We analyzed the proteomic differences between *Smu_opuB* and UA159, with a focus on the proteins involved in DNA repair mechanisms under acidic stress. One notable protein that showed differential expression is DNA polymerase I (POLI), an error-prone DNA polymerase involved in translesion synthesis and DNA repair [26,27].

POLI was significantly upregulated in the *Smu_opuB* MVs under acidic conditions (pH 5.5), suggesting that this protein plays a crucial role in the response to acidic stress. POLI is known for its ability to bypass DNA lesions during replication, although with lower fidelity compared to other DNA polymerases, making it highly mutagenic and contributing to genomic instability [27,28]. POLI is involved in repairing DNA lesions when other polymerases fail to perform the repair, by incorporating nucleotides opposite the damage site and relying on other polymerases like polymerase zeta (Pol ζ) to extend the nascent DNA chain [29]. The upregulation of POLI in *Smu_opuB* under acidic stress, implies that this strain may experience more significant DNA damage compared to UA159, or it may have a stronger DNA repair response, enhancing its resistance to acidic environments. Our proteomic analysis suggest that the latter explanation is more likely, with *Smu_opuB* having a more robust self-repair mechanism. The increased presence of POLI could be indicative of an adaptive mechanism that helps *S. mutans* survive under stress conditions by promoting error-prone repair processes.

Furthermore, the proteomic analysis in this study revealed a critical role played by *opuB* in regulating key metabolic pathways, particularly glycolysis and gluconeogenesis, in *S. mutans*. Under acidic conditions, we observed upregulation of proteins involved in glycolysis/gluconeogenesis, with a total of 16 proteins significantly affected. This suggests that *Smu_opuB* plays a key role in regulating the direction of glycolysis, which is essential for energy production and metabolic adaptation during acidic stress. Under acidic conditions, *S. mutans* faces significant challenges as the accumulation of protons lowers the pH of its environment and disrupts cellular homeostasis. To cope with this stress, the bacterium enhances its glycolytic activity, which promotes ATP production and maintains cellular function under low pH conditions [21].

While this study identifies *opuB*’s role in MV biogenesis, the pathogenic implications of MV compositional changes (*S. mutans*–host interactions) and precise regulatory mechanisms require further investigation using advanced biofilm models.

## 5. Conclusions

The *opuB* gene regulates the biogenesis, composition, and function of MVs in *S. mutans* in response to environmental pH. These findings have important implications for targeting MV-related processes to mitigate the cariogenicity of *S. mutans*.

## Figures and Tables

**Table 1:** Bacterial strains and plasmid used in this study.

Strain or Plasmid	Description	Source of Reference
UA159	*S. mutans* UA159	ATCC 700610
*Smu_opuB*	*S. mutans* UA159 with in-frame replacement with an erythromycin cassette	This study

## Data Availability

The data presented in this study are openly available in ProteomeXchange iProX at http://www.proteomexchange.org (accessed on 13 February 2025), reference number PXD060748.

## Supplementary Materials

The following supporting information can be downloaded at: https://www.mdpi.com/article/10.3390/microorganisms13040884/s1, Table S1: Strain and Primer Sequences Used in the Preparation of *Smu_opuB*. Table S2: The top 20 most significantly altered MV proteins under pH7.5 condition. Table S3: The top 20 most significantly altered MV proteins under pH5.5 condition. Figure S1: Sequencing results of positive monoclonal. Figure S2: Growth curves of *S. mutans* UA159 and *Smu_opuB* under acidic (pH 5.5) and neutral (pH 7.5) conditions. Figure S3: TEM images of MVs produced by *Smu_opuB* and UA159 under neutral (pH 7.5) and acidic (pH 5.5) conditions. Figure S4: Chain length analysis of some lipids in UA159 and *Smu_opuB* MVs under neutral conditions. Figure S5: Saturation analysis of some lipids in UA159 and *Smu_opuB* MVs under neutral conditions. Figure S6: Chain length analysis of some lipids in UA159 and *Smu_opuB* MVs under acidic conditions. Figure S7: Saturation analysis of some lipids in UA159 and *Smu_opuB* MVs under acidic conditions.

## Abbreviations

The following abbreviations are used in this manuscript:

*S. mutans*	*Streptococcus mutans*
MVs	Membrane vesicles
*Smu_opuB*	*opuB*-deficient strain
BHI	brain heart infusion
TEM	Transmission Electron Microscopy
LC-MS/MS	liquid chromatography–tandem mass spectrometry
GO	gene ontology
KEGG	Kyoto Encyclopedia of Genes and Genomes
PPI	protein–protein interaction
PCA	principal component analysis
POLI	polymerase I
AcCa	acylcarnitine
CerG2GNAc1	dihexosyl-N-acetylhexosyl-ceramideceramide
MG	monoglyceride
PC	phosphatidylcholine
LPE	lysophosphatidylethanolamine
LPG	Lysophosphatidylglycerol
PE	phosphatidylethanolamine
GT3	glycosyltransferase 3
