# Supplementary material for: Role of opuB in Modulating Membrane Vesicle Composition and Function in Streptococcus mutans Under Neutral and Acidic Conditions
Source: Microorganisms. 2025 Apr 11;13(4):884. doi: 10.3390/microorganisms13040884 (PMC12029584; doi:10.3390/microorganisms13040884)
Supplement: Supplementary file 1 [file microorganisms-13-00884-s001.zip › microorganisms-3537735-supplementary.pdf]

The Role of *opuB* in Modulating Membrane Vesicles Composition and Function in *Streptococcus mutans* under Neutral and Acidic Conditions

Wenyu Wang [1,2], Yiyi Huang [1,2] and Huangcai Lin [1,2,*]and Yina Cao [1,2,*]

[1]Hospital of Stomatology, Guanghua School of Stomatology, Sun Yat-sen University, Guangzhou, China

[2]Guangdong Provincial Key Laboratory of Stomatology, Sun Yat-sen University, Guangzhou, China

* Corresponding Author: Huancai Lin and Yina Cao

Postal address: Guangzhou, Guangdong, 510000, China

E-mail addresses: Huancai Lin (linhc@mail.sysu.edu.cn) or Yina Cao (caoyn5@mail.sysu.edu.cn)

Supplementary Information

Preparation of *Smu_opuB*

Supplementary Table

Table S1: Strain and Primer Sequences Used in the Preparation of *Smu_opuB*

Table S2: The top 20 most significantly altered MV proteins under pH7.5 condition

Table S3: The top 20 most significantly altered MV proteins under pH5.5 condition

Erythromycin resistance gene (738 bp)

Supplementary Figure

Figure S1. Sequencing results of positive monoclonal.

Figure S2. Growth curves of *S. mutans* UA159 and *Smu_opuB* under acidic (pH 5.5) and neutral (pH 7.5) conditions.

Figure S3. TEM images of MVs produced by *Smu_opuB* and UA159 under neutral (pH 7.5) and acidic (pH 5.5) conditions.

Figure S4. Chain length analysis of some lipids in UA159 and *Smu_opuB* MVs under neutral conditions.

Figure S5. Saturation analysis of some lipids in UA159 and *Smu_opuB* MVs under neutral conditions.

Figure S6. Chain length analysis of some lipids in UA159 and *Smu_opuB* MVs under acidic conditions.

Figure S7. Saturation analysis of some lipids in UA159 and *Smu_opuB* MVs under acidic conditions.

.

Preparation of ***Smu_opuB***

1. Bacterial culture and extraction of bacterial genomic DNA (gDNA)

1) The UA159 strain stored at -80 °C was revived in BHI medium. After morphological and biochemical identification, the plate was stored at 4 °C for short-term seed preservation.

2) A single clone was picked from the plate and inoculated into BHI liquid medium for overnight culture.

3) The overnight culture solution was extracted from the bacteria according to the instructions of the bacterial genomic DNA extraction kit.

4) The purity and concentration were determined and stored at -20 °C.

2. PCR amplification and connection of target fragments

1) PCR was used to amplify the fragments up, erm and down. The reaction conditions were 94 °C pre-denaturation for 3 min; 94 °C denaturation for 30 s, 55 °C annealing for 30 s, 72 °C extension for 1 min, 30 cycles, and 72 °C extension for another 5 min. The reaction was terminated at 4 °C.

2) 1% agarose gel electrophoresis was used to identify the PCR amplification product, and the gel was recovered and the product concentration and purity were determined, and stored at -20 °C.

3) Using up, erm, and down as templates, upF and dnR as primers, fusion PCR was used to connect the above three fragments (recorded as up-erm-dn, with a size of about 1.6 kb). The reaction conditions were 94 °C pre-denaturation for 3 min; 94 °C denaturation for 30 s, 55 °C annealing for 30 s, 72 °C extension for 2 min, 30 cycles; 72 °C extension for another 5 min. The reaction was terminated at 4 °C.

4) 1% agarose gel electrophoresis was used to identify the PCR amplification product, and stored at -20 °C.

3. Transformation and screening

1) Pick UA159 monoclonal clones and inoculate them in BHI liquid medium. Take the overnight culture solution and dilute it in BHI medium at 1:20 and culture it for 2-3 h until the bacterial solution OD_{600} = 0.2-0.3.

2) Take bacterial solution and add samples according to the following groups: group a: 500 μL bacterial solution + 5 μL up-erm-dn + 0.5 μL CSP (1 μg·μL-1); group b: 500 μL bacterial solution + 0.5 μL CSP (1 μg·μL^{-1}); group c (negative control): 500 μL bacterial solution. After adding and mixing, incubate in a 37℃ incubator for 2 h in microanaerobic culture.

3) Take 200 μL of bacterial solution from groups a, b, and c and spread them on BHI agar plates containing 12.5 μg·mL^{-1} erythromycin, and incubate in a 37℃ incubator for 48 h in microanaerobic culture.

4) Send the obtained positive monoclonal clones to the Guangzhou Branch of Beijing Liuhe BGI Gene Technology Co., Ltd. for sequencing.

Table S1: Strain and Primer Sequences Used in the Preparation of *Smu_opuB*

**Strain**	**Source**
S. mutans UA159 （ATCC®700 610™）	Guangdong Microbiology Research Institute Culture Collection Center
**Primer Name**	**Nucleotide Sequence (5’-3’)**
upF	CGCGACTGTCAGAAAAGGAG
up-erm	TATTTTATATTTTTGTTCATCTTTATTCC TCTTTTCTAAT
ermF	ATGAACAAAAATATAAAATATTCTCA
ermR	TTATTTCCTCCCGTTAAATAAT
dnF-erm	ATTATTTAACGGGAGGAAATAAGAATA AGGAGGTTAGAACAC
dnR	ATCAAAAATTTCACTGCGGA

Erythromycin resistance gene (738 bp)

ATGAACAAAAATATAAAATATTCTCAAAACTTTTTAACGAGTGAAAAAGTA
CTCAACCAAATAATAAAACAATTGAATTT
AAAAGAAACCGATACCGTTTACGAAATTGGAACAGGTAAAGGGCATTTAA
CGACGAAACTGGCTAAAATAAGTAAACAGG
TAACGTCTATTGAATTAGACAGTCATCTATTCAACTTATCGTCAGAAAAATT
AAAACTGAACATTCGTGTCACTTTAATT
CACCAAGATATTCTACAGTTTCAATTCCCTAACAAACAGAGGTATAAAATTG
TTGGGAGTATTCCTTACCATTTAAGCAC
ACAAATTATTAAAAAAGTGGTTTTTGAAAGCCATGCGTCTGACATCTATCTG
ATTGTTGAAGAAGGATTCTACAAGCGTA
CCTTGGATATTCACCGAACACTAGGGTTGCTCTTGCACACTCAAGTCTCGAT
TCAGCAATTGCTTAAGCTGCCAGCGGAA
TGCTTTCATCCTAAACCAAAAGTAAACAGTGTCTTAATAAAACTTACCCGCC
ATACCACAGATGTTCCAGATAAATATTG
GAAGCTATATACGTACTTTGTTTCAAAATGGGTCAATCGAGAATATCGTCAA
CTGTTTACTAAAAATCAGTTTCATCAAG
CAATGAAACACGCCAAAGTAAACAATTTAAGTACCATTACTTATGAGCAAG
TATTGTCTATTTTTAATAGTTATCTATTA
TTTAACGGGAGGAAATAA

```
UA159     1    CTCTTCTAGCAGGACTTGACAGTCCTAAAAAAGGAAAAATCCTTCTTAATAATCAAAATATTAGTGAAAA 70
Smu_opuB  1    CTCTTCTAGCAGGACTTGACAGTCCTAAAAAAGGAAAAATCCTTCTTAATAATCAAAATATTAGTGAAAA 70

UA159     71   AGGCTTAACTGATTATCGTAAAAATGCTGTCTCAACCATTTTCCAATCCTATAATCTTTTGCCTTATATG 140
Smu_opuB  71   AGGCTTAACTGATTATCGTAAAAATGCTGTCTCAACCATTTTCCAATCCTATAATCTTTTGCCTTATATG 140

UA159     141  ACAGCTCAACAAAATGTTGAAACCGCACTTGATATTTCATCTGTCAAGACAACAAGTGAAAAAATTGTAA 210
Smu_opuB  141  ACAGCTCAACAAAATGTTGAAACCGCACTTGATATTTCATCTGTCAAGACAACAAGTGAAAAAATTGTAA 210

UA159     211  CATTATTTGATAATGTTGGTATTTCTGAAGATTTGATAGATAAACCTGTCTTACATCTCTCCGGAGGTCA 280
Smu_opuB  211  CATTATTTGATAATGTTGGTATTTCTGAAGATTTGATAGATAAACCTGTCTTACATCTCTCCGGAGGTCA 280

UA159     281  GCAACAAAGAGTTGCTATCGTGAGGGCGCTCGCTAGCGAACACAATATCATTATTGCTGATGAACCAACT 350
Smu_opuB  281  GCAACAAAGAGTTGCTATCGTGAGGGCGCTCGCTAGCGAACACAATATCATTATTGCTGATGAACCAACT 350

UA159     351  GGTAATCTAGACGAAACAACAACCAAAGATATTGTTACTATTTTTAAAAAGATTGCCCATGAGCAGAAGA 420
Smu_opuB  351  GGTAATCTAGACGAAACAACAACCAAAGATATTGTTACTATTTTTAAAAAGATTGCCCATGAGCAGAAGA 420

UA159     421  AAACGGTGATTATTGTTACGCATGAACGAGAAGTTGCTGATAGTTCTGATATTATTTTCGAATTACGCAA 490
Smu_opuB  421  AAACGGTGATTATTGTTACGCATGAACGAGAAGTTGCTGATAGTTCTGATATTATTTTCGAATTACGCAA 490

UA159     491  AAAAGAATTTACATTAATTTAAAAGCAAGCGGTGGTTTTCTTTTCGCCGCTTGCTTTTTACTAAGTCAGA 560
Smu_opuB  491  AAAAGAATTTACATTAATTTAAAAGCAAGCGGTGGTTTTCTTTTCGCCGCTTGCTTTTTACTAAGTCAGA 560

UA159     561  TTGTTTCTTGTATGCTTGCATTCTTTTAGCATAAATATTAGTTTCAATTTTTTGAGCTCTTTTTAGGTTT 630
Smu_opuB  561  TTGTTTCTTGTATGCTTGCATTCTTTTAGCATAAATATTAGTTTCAATTTTTTGAGCTCTTTTTAGGTTT 630

UA159     631  TTATTTCAGCAAACCTGTTTTTTTGAGATAATGATGAGCTACTGTTTTAGCAGATTTTCCTTTGACTTTA 700
Smu_opuB  631  TTATTTC-------------------------------CTCC-----------------------CGTTA 646

UA159     701  ACCTGATAATTCATGTCGCTCATTTGATTTTCTGTAATTTTTCCAGCTAAGCGATTCAGAATCTTTTTTA 770
Smu_opuB  647  A----ATAATTGAT-----------------------------------AACTATTAAAAA-------TA 670

UA159     771  GTTCAGGATGTTTTTGTAACAATTTCTTTTTGAATAAAGGTGCTCCCTGATAGGGTGGGAAAAGATTTTT 840
Smu_opuB  671  G-----------------ACAAT--------------ACTTGCTC----ATA------------------ 687

UA159     841  GTTATCCTTGAGAGTAACCAAGTCGTATTCCTTGATTTCTGAATCTGTTGA-ATAGGCGTCAGTGATGTC 909
Smu_opuB  687  ------------AGTAAC-----GGTA-----------CTTAAATTGTTTACTTTGGCGT--GT------ 721

UA159     910  AATATCACCACTCTTAATTGCTTGATAGCGCAAAGCAGGTTCCATTGTACTCACCTGTAAATTAAGTCCA 979
Smu_opuB  721  -------------TTCATTGCTTGAT---GAAA---------------------CTG---ATT------- 744

UA159     980  TAAAGACTCTTCAATCCTTTGTTTCCATCTTTTCGGTCATTAAATTCGAGAGTAAATCCTGCTTTTGCTG 1049
Smu_opuB  744  -----------------TTT------------------------------AGTAAA-------------- 753

UA159     1050 TATTTTCAACCTTTTTCAAATCAGAAATAGACTTTAATCCTTTTTCTTTAGCAAATGATTTCTTAACAGC 1119
Smu_opuB  753  ---------------------CAG---TTGAC-------------------------------------- 761

UA159     1120 TACAGCATAGGTATTTTGATATT-TCATGGGCTTTAGATAGACAAGATTGTCCTGTTTTAAAATACCATC 1188
Smu_opuB  761  -----------------GATATTCTC-------------------GATTGACCCATTTTGAAACA----- 790

UA159     1189 ACGTGCTTCTTCGTAAACTGTTTGAGGATTATTGGACGT-TTTAGGTGGATTTTTTAGAAGACTACTTGT 1257
Smu_opuB  790  --------------AA-----------------GTACGTATATAG--------------------CTTCC 809

UA159     1258 AATTGTTCCAGTAAACTCTGGGTAAATATCAATATCACCTTTTTTAGGGCTTCATAAAGAAAGGTTGTT 1327
Smu_opuB  810  AATATTT--------ATCTGG---AACATC----------TGTGGTATGGC------------GG----- 841
```

```
UA159     1328 TTTCCAAAATTAGGTTTGACTTTTACTGATAGGTGACTATCTTGCTCAATTAATTCTTTATACATATTAA 1397
Smu_opuB  841  ---------GTAAGTTT-----------------------------------------------TATTAA 855

UA159     1398 TCAGGACTTCTGGTTCAGGTCCCATTTTACCAGCAATGACAATTTCTTTCTGACTCTGACTGGTTTTAAT 1467
Smu_opuB  855  ----GAC-ACTG-------------TTTAC------------TTT---------------TGGTTT---- 876

UA159     1468 CACATTTGGAAGATAAGAGGCGCCCAAACCAATCAACATGATAAAAAAGGCAAGCATAATCTTTTTCAGC 1537
Smu_opuB  876  -----------------AGG-----------------ATGA----------AAGCA--------TTCCGC 894

UA159     1538 GAAGCTTTTTCTAAAAACTTAATAGCAAGATTAAAAAGAATAGCTAAAAGAGCTGAAGAAATAGCTCCTA 1607
Smu_opuB  894  ----------------------TGGC------------------------AGCTTAAGCAATTGCT---- 914

UA159     1608 TTAAAATCAGAGA----AGTGTTGTTACGATCAATTCCAAGAAGAATGAAGGAACCCAACCCTCCAGCTC 1673
Smu_opuB  914  ---GAATC-GAGACTTGAGTG--------------TGCAAGA-------------GCAACCCT--AG--- 948

UA159     1674 CAATCAAGGCTGCCAAGGTCGCTGTCCCAATAATCATAACAGCCGCTGTCCTTACTCCAGAAATGATAAC 1743
Smu_opuB  948  ---------------TGTTCGGTG----AATA-----------------------TCCA----------- 965

UA159     1744 TGGCATAGCCAGCGCTAATTCAAATTTTTTCAGT-CGCTCCCACTTGGTCATACCGAAAGCTTGACCAGC 1812
Smu_opuB  965  ------AG------------------------GTACG------CTTG----TA--GAATCCTT------C 987

UA159     1813 TTCGACCAAACTTGGGTCA-ATAGTTGTTAGACCAGTGATGGTATTTTGCAAAATTGGAAAAAGGGCATA 1881
Smu_opuB  988  TTCAACAA--------TCAGATAGATGTCAGAC--------------------------------GCAT- 1016

UA159     1882 GATAACCAGAGCCGCTACGGCTGGCACCGTTCCAATCCCCATAAAAGGGATGAATAAGCCTAAAAGAGCC 1951
Smu_opuB  1016 ------------------GGCT-------TTC-------------------------------------- 1023

UA159     1952 AGAGAAGGAATCGTTTGGAAAATCC-CTGAAATTTGTAAAATAATCTCTGCTATTCTCTTTCGATGACTG 2020
Smu_opuB  1023 ------------------AAAAACCACT----TTTTT--AATAATTTGTG--------------TG-CTT 1054

UA159     2021 ATAAAGATAGCCAGAGGAACAGCAAGGATAATGGCTATTAAAAGTGACAATAAAGATATTTGTAAATGCT 2090
Smu_opuB  1055 A----------------------------AATGG----------------TAAGG---------AATACT 1071

UA159     2090 -CTAACAAGGACTGTGTCCAGTCAGCAAAGCGATTTTGGAAGGTTGTCATTAAATCGGTCATTAGTTATC 2159
Smu_opuB  1072 CCCAACA-------------------------ATTTT--------------------------------- 1083

UA159     2160 ACCTCCAAATAAATCTGCCACAAAATCATTGACCGGATTTTGCGCCATATCTTTAGGACTAGCCAATTGA 2229
Smu_opuB  1083 --------ATACCTCTG---------------------TTTG----------TTAGG-----------GA 1103

UA159     2230 ATAATCCGCCCCTTTTGAAA-AATAGCAATTCGATCTGCTAACTTTTTGGCTTCTTCAATATCATGCGTT 2298
Smu_opuB  1104 A-------------TTGAAACTGTAG-AAT---ATC----------TTGG-----TGAAT-TAA--AGTG 1138

UA159     2299 ACAAAAACAACTGTCATTCCTAATTCTTTTTGTAAAGAGAGTGTCAAATCTTGTAGTTGTTTTCTCGAAA 2368
Smu_opuB  1139 ACACGAGCA---TTCAGTTTTAA---TTTTTCTGACGATA--------------AGTTG---------AA 1179

UA159     2369 TAGGATCCAGGGCTGAAAAAGGCTCGTCCATTAAAAGAATATGGGGATTTGCAATAATGGCACGTAGAAT 2438
Smu_opuB  1180 TAG-----ATGACTG--------TC------TAA---------------TTCAATA----GACGT----- 1206

UA159     2439 ACCTACCCGTTGTTGCTCACCACCTGATAGGTCACGTGGATAACGTTCCAAATACTTATCTGCTGGCAAG 2508
Smu_opuB  1206 ---TACCTGTT-----------------------------------------TACTTATTT------TAG 1226

UA159     2509 CCAGCTTTATCAAGTAATTTCTTCGTTTTTTCGAGTCGATCTGCTTTTGACCAAGCCTTCATTTCAGGAA 2578
Smu_opuB  1227 CCAG-------------TTTCGTCGTT-----------AAATGCCCTTTACC------------------ 1254

UA159     2579 TCAATTCAATATTTTCTCTGACAGTCAGATTGGGGAAAAGAGCAATTTGCTGAAGAACATAACCCATATC 2648
Smu_opuB  1254 -----------TGTTC--------------------------CAATTT---------CGTAAACGGTATC 1278
```

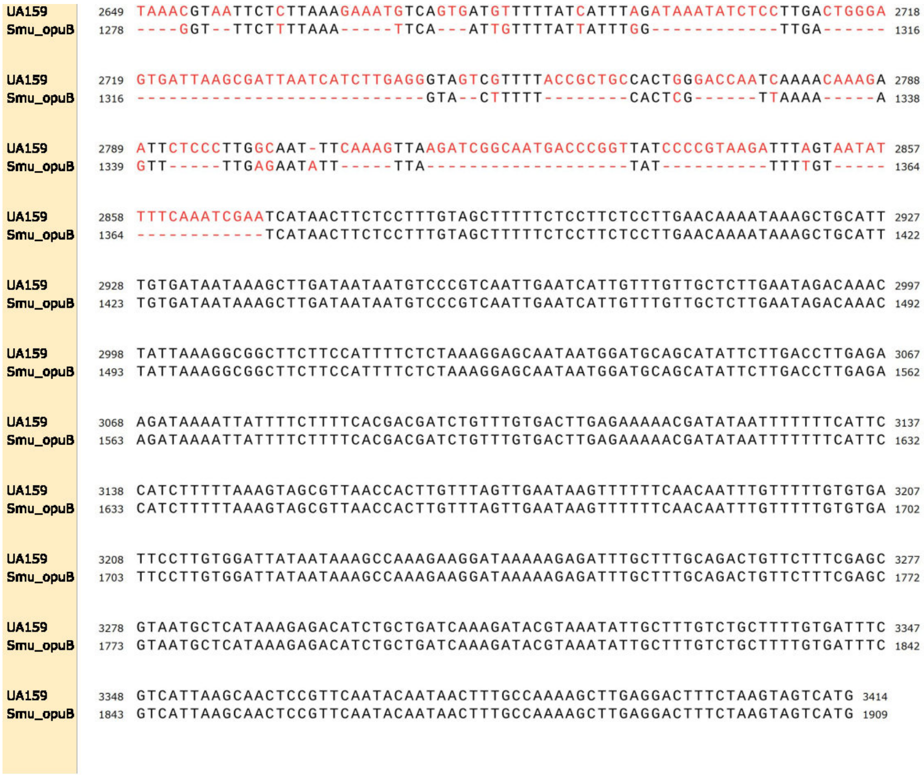

Figure S1. Sequencing results of positive monoclonal.

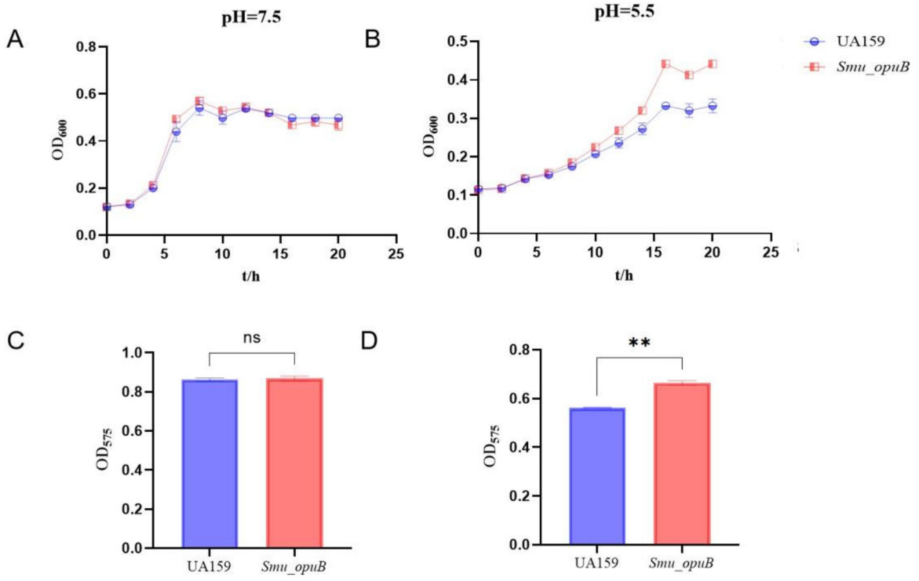

Figure S2. Growth and biofilm formation of *Smu_opuB* and UA159 under acidic and neutral conditions. Growth curves of *Smu_opuB* and UA159 under (A) neutral and (B) acidic conditions. Biofilm formation of *Smu_opuB* and UA159 after 24 hours under (C) neutral and (D) acidic conditions., assessed by crystal violet staining.

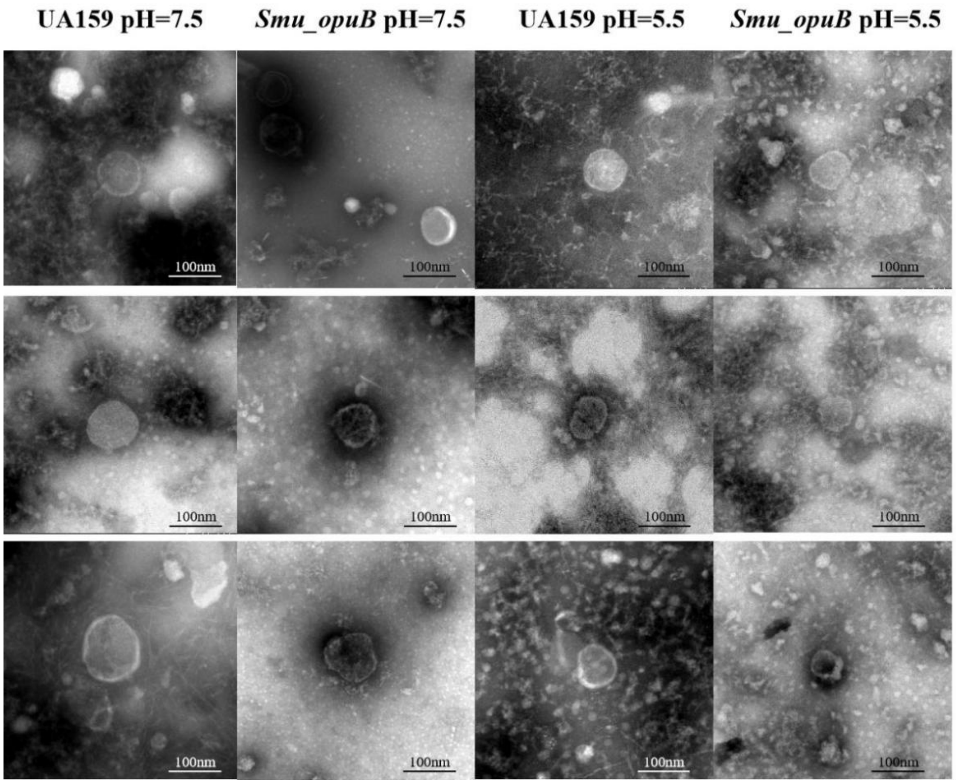

Figure S3. TEM images of MVs produced by *Smu_opuB* and UA159 under neutral (pH 7.5) and acidic (pH 5.5) conditions. For each group, three representative images are shown to provide a more comprehensive view of MV morphology. Scale bars are indicated in each image.

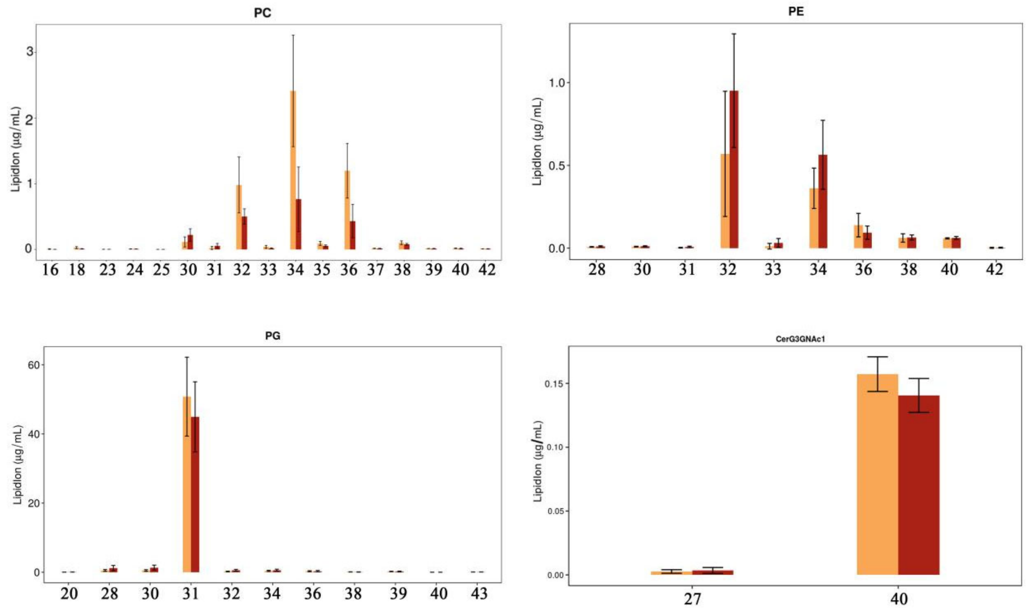

Figure S4. Chain length analysis of some lipids in UA159 and *Smu_opuB* MVs under neutral conditions. n=6. Data for all panels are mean ± S.D. *P < 0.05 (Student's *t*-test).

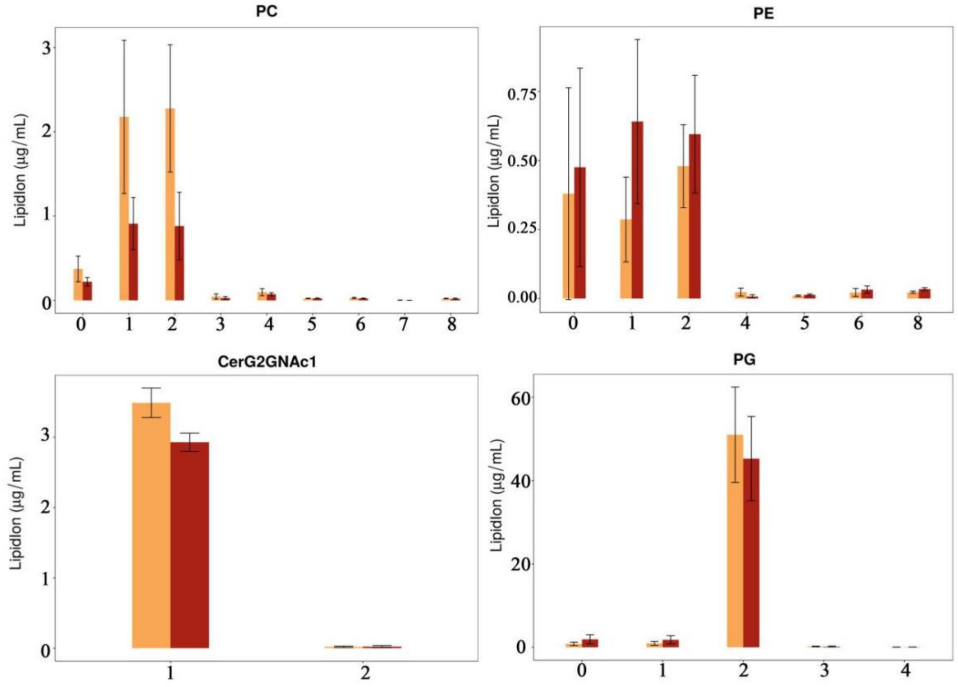

Figure S5. Saturation analysis of some lipids in UA159 and *Smu_opuB* MVs under neutral conditions. n=6. Data for all panels are mean ± S.D. *P < 0.05(Student's *t*-test).

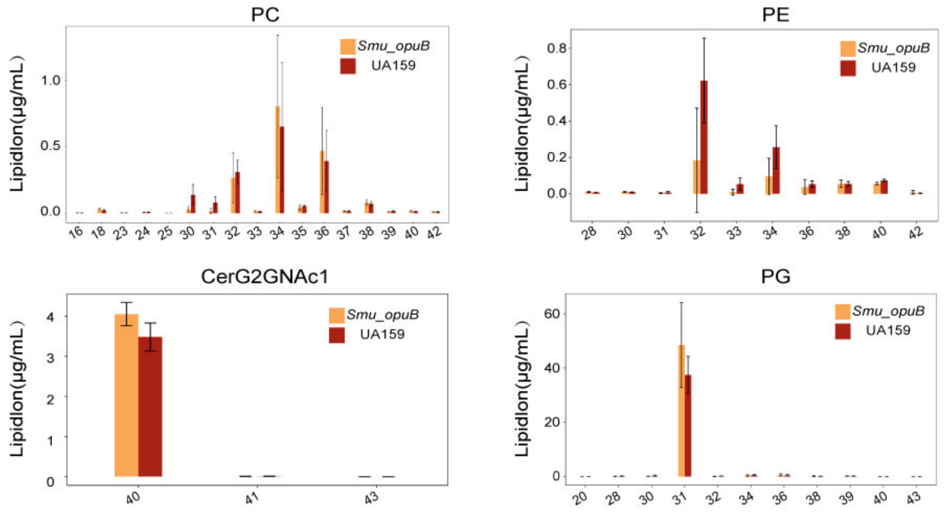

Figure S6. Chain length analysis of some lipids in UA159 and *Smu_opuB* MVs under acidic conditions. n=6. Data for all panels are mean ± S.D. *P < 0.05 (Student's *t*-test).

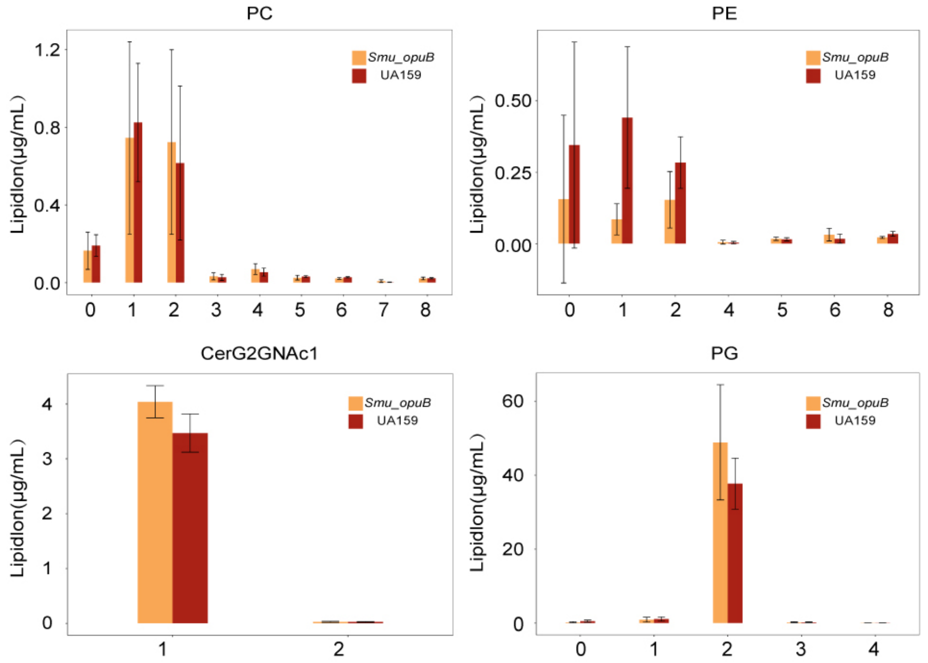

Figure S7. Saturation analysis of some lipids in UA159 and *Smu_opuB* MVs under acidic conditions. n=6. Data for all panels are mean ± S.D. *P < 0.05 (Student's *t*-test).

Table S2 The top 20 most significantly altered MV proteins under pH7.5 condition

Protein Name	Gene Name	MolWeight	*Smu_opuB*/UA159	*p* value
Malonyl-CoA acyl-carrier-protein transacylase	*SMU_1344c*	47.086	40.696	0.019
rRNA adenine N-6-methyltransferase	*erm(B)*	28.795	34.575	0.036
DUF1307 domain-containing protein	*A6J86_004325*	19.683	27.666	0.018
Glucosamine-6-phosphate deaminase	*nagB*	25.473	19.354	0.040
HNH endonuclease	*SMU_1205c*	19.542	19.091	0.042
ABC transporter, amino acid binding protein	*SMU_459*	30.509	18.059	0.026
AgI/II	*spaP*	169.97	16.877	0.031
50S ribosomal protein L15	*rplO*	15.446	13.528	0.042
Dextranase	*dexA*	94.481	12.798	0.037
30S ribosomal protein S11	*rpsK*	13.399	12.751	0.042
(R)-2-hydroxyglutaryl-CoA dehydratase activator-related protein	*SMU_438c*	159.32	0.125	0.005
Elongation factor Ts	*tsf*	37.719	0.100	0.037
Amino acid ABC transporter, ATP-binding protein	*SMU_936*	28.357	0.095	0.041
ABC sugar transporter, permease protein	*SMU_1118c*	33.775	0.088	0.024
Ribosomal protein L11 methyltransferase	*prmA*	34.543	0.083	0.004
Metal-dependent transcriptional regulator	*sloR*	25.076	0.083	0.000
Cytoplasmic protein	*SMU82_02671*	24.169	0.079	0.042
ABC transporter, ATP-binding protein	*SMU_923*	64.59	0.066	0.001
Oxidoreductase	*SMU_728*	32.188	0.064	0.006
ABC transporter, ATP-binding protein	*SMU_922*	66.968	0.039	0.002

Table S3 The top 20 most significantly altered MV proteins under pH5.5 condition

Protein Name	Gene Name	MolWeight	*Smu_opuB*/ UA159	p value
Probable transcriptional regulatory protein SMU82_03911	SMU82_03911	25.74	34.332	0.005
rRNA adenine N-6-methyltransferase	erm(B)	28.795	33.964	0.002
GTP cyclohydrolase 1 type 2 homolog	SMU_1463c	29.74	17.912	0.002
Acetolactate synthase AlsS	A6J86_004270	60.758	16.534	0.000
Ribulose-phosphate 3-epimerase	rpe	23.722	15.402	0.000
Glucosamine-6-phosphate deaminase	nagB	25.473	13.578	0.000
Histidinol dehydrogenase	hisD	46.45	12.250	0.000
Shikimate kinase	aroK	17.612	11.323	0.022
Ribonucleoside-diphosphate reductase subunit beta	nrdG	36.788	11.226	0.004
67 kDa myosin-crossreactive streptococcal antigen-like protein	SMU_1584c	67.347	11.034	0.000
Hydrolase	SMU_1171c	29.399	0.120	0.028
Transcriptional repressor	A6J86_008015	18.212	0.117	0.016
ABC transporter, ATP-binding protein	SMU_1078c	65.369	0.094	0.001
ABC transporter, ATP-binding protein	SMU_922	66.968	0.088	0.036
Homoserine O-acetyltransferase	metAA	36.746	0.063	0.022
Integral membrane protein	SMU_237c	45.139	0.057	0.035
Phosphate transport system permease protein PstA	pstC	31.773	0.043	0.021
UPF0246 protein SMU82_04358	SMU82_04358	28.04	0.041	0.022
Mutans bacitracin-resistance related protein B	mbrB	75.823	0.038	0.028
Cell division protein FtsL	ftsL	12.298	0.026	0.000
